# Statistical modelling predicts almost complete loss of major periglacial processes in Northern Europe by 2100

**DOI:** 10.1038/s41467-017-00669-3

**Published:** 2017-09-11

**Authors:** Juha Aalto, Stephan Harrison, Miska Luoto

**Affiliations:** 1 Department of Geosciences and Geography, University of Helsinki, P.O. Box 64, Gustaf Hällströmin katu 2a, 00014 Helsinki, Finland; 2 Finnish Meteorological Institute, P.O. Box 503, FI-00101 Helsinki, Finland; 3 College of Life and Environmental Sciences, University of Exeter, Penryn, TR10 9EZ UK

## Abstract

The periglacial realm is a major part of the cryosphere, covering a quarter of Earth’s land surface. Cryogenic land surface processes (LSPs) control landscape development, ecosystem functioning and climate through biogeochemical feedbacks, but their response to contemporary climate change is unclear. Here, by statistically modelling the current and future distributions of four major LSPs unique to periglacial regions at fine scale, we show fundamental changes in the periglacial climate realm are inevitable with future climate change. Even with the most optimistic CO_2_ emissions scenario (Representative Concentration Pathway (RCP) 2.6) we predict a 72% reduction in the current periglacial climate realm by 2050 in our climatically sensitive northern Europe study area. These impacts are projected to be especially severe in high-latitude continental interiors. We further predict that by the end of the twenty-first century active periglacial LSPs will exist only at high elevations. These results forecast a future tipping point in the operation of cold-region LSP, and predict fundamental landscape-level modifications in ground conditions and related atmospheric feedbacks.

## Abstract (web summary)

Cryogenic land surface processes characterise the periglacial realm and control landscape development and ecosystem functioning. Here, via statistical modelling, the authors predict a 72% reduction of the periglacial realm in Northern Europe by 2050, and almost complete disappearance by 2100.

## Introduction

Periglacial environments with frost-induced and permafrost-controlled land surfaces processes (LSPs) are vital components of the cryosphere^1, 2^. With current Arctic amplification of climate warming^3^, substantial alterations in this sensitive and important area of the Earth’s system are already observed^2^, including glacier recession^4^, shrub expansion to alpine tundra^5^ and changes in permafrost thermal–hydrological regimes^6^. Importantly, these changes in ground conditions modify, among others, biogeochemical cycles (e.g., terrestrial CO_2_ and CH_4_) and reflectance (i.e., albedo) triggering climate feedbacks^7–9^. Thus, better understanding of the response of the periglacial climate realm to climate change is critical for assessing climate change mitigation, and extensive modelling studies at various geographical scales are urgently required^7^.

**Fig. 1:**
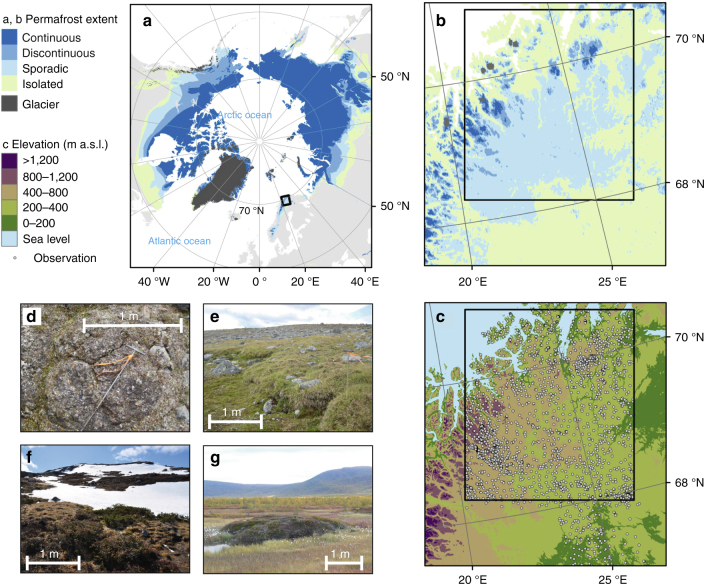
The location of the study domain and LSP observation sites in northernmost Europe. **a**, **b** The study area in relation to the circum-Arctic extent of permafrost^23, 24^ indicated as: continuous=90–100% of the area covered by permafrost, discontinuous=50–90%, sporadic=10–50% and isolated=0–10%, respectively. **c** The observation locations (*n* = 2,917) and the relief of the study area. *Black rectangles* in **b**, **c** depict the model prediction domain. Photos show examples of typical surface features of cryogenic land surface processes (scales are only directive): cryoturbation (**d**; small-scaled polygonal patterned soil) gelifluction (**e**; gelifluction lobes), nivation (**f**, snow accumulation sites) and permafrost mounding (**g**; palsas), respectively. Photos by J.A. and M.L.

The combined spatial extent of active cryogenic LSPs constitutes the periglacial climate realm^1^. This prevails across high latitudes and elevations, at present covering ca. 25% of the Earth’s terrestrial areas. Here, LSPs create surface geomorphological features which are unique to periglacial regions including patterned ground and hummocky terrain associated with cryoturbation, gelifluction terraces and lobes, nivation features associated with erosion by snow patches and palsa mires which develop through permafrost mounding^10^ (Fig. 1). Periglacial LSPs play a crucial role by controlling denudation processes^11^, vegetation community structure and productivity^12, 13^, hydrology^14^ and biogeochemical cycles^7, 15, 16^. Currently, the response of periglacial LSPs to climate warming is highly uncertain. Although a rapid response is expected^7^, because the broad-scale distribution of cryogenic ground processes is coupled with climatic gradients^17, 18^ and often, but not necessarily, with the presence of permafrost^1, 19^, the details of this and timing are lacking. This strong LSP–climate response^17, 20^ is locally modified by lithology and edaphic (reflecting, e.g., glaciation heritage)^1, 11^ and topographical characteristics^17, 21, 22^. For example, gelifluction operates on inclined surfaces with frost-susceptible fine-grained soils, while cryoturbation features are common in flat valley bottoms and mountain tops^1, 17^. The development of palsa mires through permafrost mounding is expected to occur on open low-elevation peat lands where strong winds redistribute snow allowing for a deep frost penetration^10, 18^.

Here, we use remotely sensed and field-quantified data of LSPs at an unprecedented scale focusing on active surface features related to cryoturbation, gelifluction, nivation and permafrost peat mounding to investigate the current and future extent of the periglacial climate realm across a high-latitude Fennoscandia region of ca. 78,000 km^2^ (Fig. 1). We argue that the absence of deep permafrost (compared with, e.g., High Arctic Canada and Siberia)^23, 24^ means that in general the thermal response of LSPs to future climate change is likely to be rapid (perennial ground ice enhances soil-ambient air decoupling^25^), and thus the region is representative of environments that are especially sensitive to climate change^26^. Similar sensitive landscapes are expected to prevail across broad high-latitude areas of discontinuous and isolated permafrost, including large parts of Canada and Russia between 55 and 70° N latitudes. We relate the current occurrence of LSPs with climatic variables of freezing and thawing degree days (FDD and TDD, respectively), water and snow precipitation, local topography (potential radiation, slope angle and topographic wetness) and soil characteristics (peat and rock cover)^17^. We use an ensemble modelling approach, where methodology-related uncertainty can be controlled by merging predictions from multiple statistical algorithms (regression and machine learning) to a single agreement map^27^ (spatial resolution 50 m × 50 m; refer to Methods for a description of data compilation and statistical analysis). After investigating the baseline distributions (i.e., the current climate of 1981–2010) of the LSPs, we develop climate projections forced by three Representative Concentration Pathway (RCP) scenarios^28^ (2.6, 4.5 and 8.5, roughly equal to CO_2_ concentrations of 490, 650, 1,370 p.p.m. by the end of this century, respectively) and two time periods (2040–2069 and 2070–2099), averaged over a large group (*n* = 23) of CMIP5 climate simulations^29^. We show potential for a notable reduction in the current periglacial climate realm in our study area, and predict that by the end of the twenty-first century active periglacial LSPs will exist only at high elevations.

## Results

### Present distributions of cryogenic LSPs

**Fig. 2:**
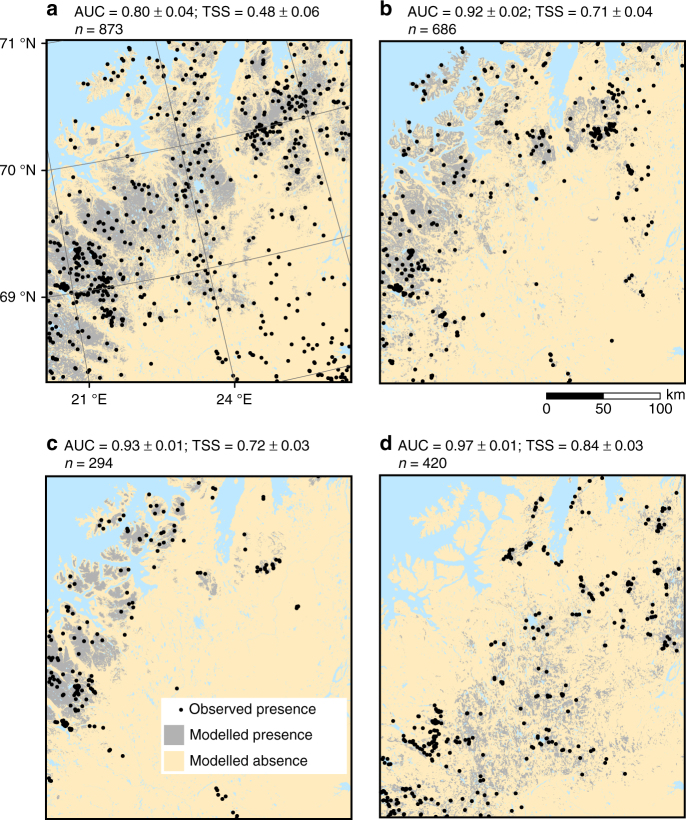
The modelled baseline occurrences of the four LSPs based on majority vote ensemble. The *n* in the title denotes the number of observed presences (*black dots*), while all the observation sites (*n* = 2,917) are presented in Fig. 1c. The modelling performance is measured as the area under the curve of a receiver operating characteristic plot (AUC) and the true skill statistics (TSS). The evaluation statistics show the mean (±s.d.) over four modelling techniques and 100 cross-validation runs conducted for each LSP (**a** cryoturbation, **b** gelifluction, **c** nivation and **d** permafrost mounding) using a random sampling procedure

**Fig. 3:**
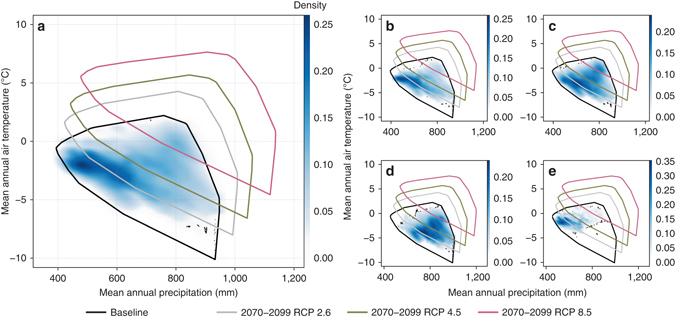
The dwindling periglacial climate. The density scatterplots represent the modelled occurrence of the LSP (*blue shades*; combined spatial extent of individual LSPs in the large plot, **a**) compared to baseline (climate of 1981–2010) mean annual air temperature and mean annual precipitation in the study area. The *black dots* indicate individual modelled LSP occurrences (**b** cryoturbation, **c** gelifluction, **d** nivation and **e** permafrost mounding) based on empirical data with total of 2,917 observations. The *polygons* depict the convex hulls (i.e., the minimum bounding boxes) of the two climate variables in the study area, and at four time periods and/or climate change scenarios, indicating the shift in climatic conditions in respect to the current periglacial climate realm of the study area

**Fig. 4:**
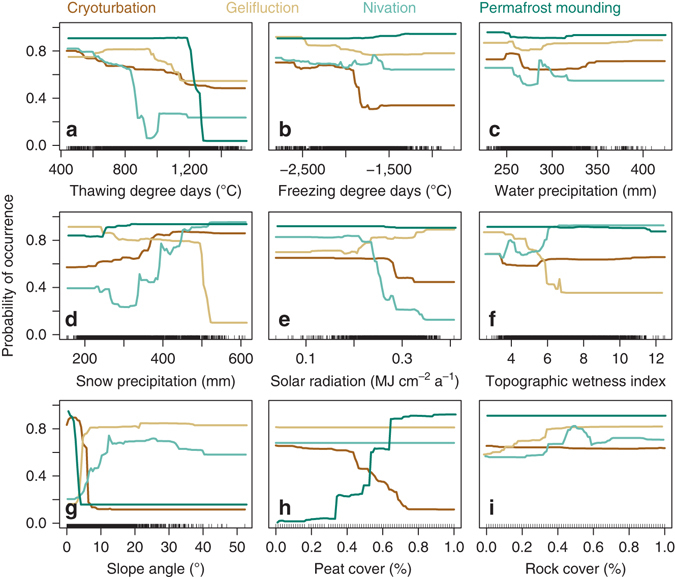
The relationships between the investigated cryogenic land surface processes and environmental predictors. Partial dependency plots estimated by generalized boosting method (GBM) depict the effect of a predictor (**a** thawing degree days, **b** freezing degree days, **c** water precipitation, **d** snow precipitation, **e** solar radiation, **f** topographic wetness index, **g** slope angle, **h** peat cover, and **i** rock cover) on LSP (indicated with coloured lines: *dark brown*=cryoturbation, *light brown*=gelifluction, *turquoise*=nivation, *dark green*=permafrost mounding) after other predictors have been fixed to their mean values

**Fig. 5:**
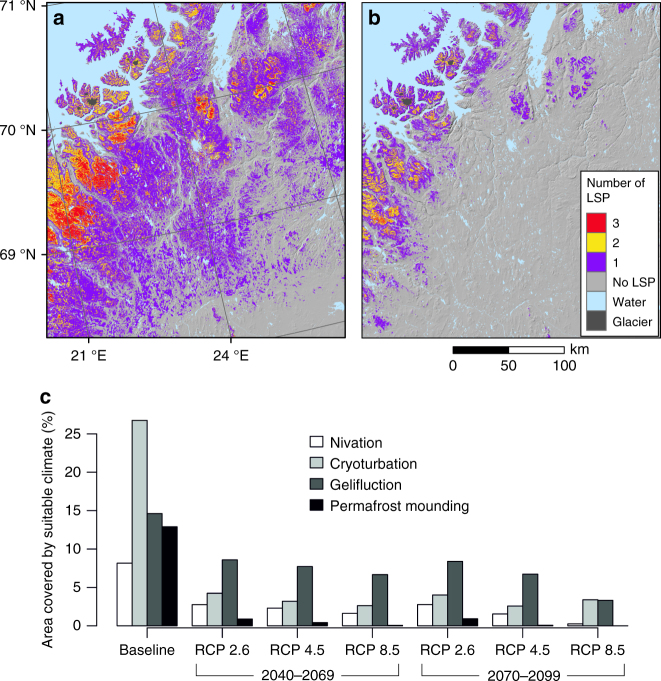
The predicted loss of conditions allowing cryogenic land surface processes. **a**, **b** The suitable overlapping conditions for the land surface processes (LSP) under baseline (i.e., current climate of 1981–2010) and future climate (2040–2069 RCP 4.5), respectively, while **c** shows the area covered by suitable climate for individual LSP at each time period and climate change scenario. The current extent of glaciers were masked out from the analysis

Our forecasts of the current LSPs show high agreement with the observations thus suggesting robust model transferability to similar environments (Fig. 2). The analysis of the current periglacial realm closely corresponds to earlier definitions^1, 20, 30^, marking mean annual air temperature (MAAT) of +2 °C as a rough upper limit for cryogenic ground processes (Fig. 3). At present, cryoturbation, gelifluction and nivation are active across a broad range of climate conditions, while permafrost mounding is most concentrated with MAAT of ~ −2 °C and low to moderate annual precipitation sum (400–600 mm) (Fig. 3). The probability of active LSPs increases towards cold air temperatures (Fig. 4). Permafrost mounding, nivation and gelifluction are highly sensitive to TDD (advancing permafrost and snow melt, and soil wetting, respectively), whereas cryoturbation is more constrained by FDD which is strongly linked to frost intensity. In turn, gelifluction is controlled by both FDD and TDD affecting frost penetration and spring melt, respectively^20^. In addition to climatic factors, the periglacial climate realm is strongly mediated by local topographical heterogeneity and soil characteristics^17^ (Fig. 4). For example, all studied LSPs are strongly linked to slope angle representing different responses (positive for gelifluction and nivation and negative for cryoturbation and permafrost mounding) to factors such as mass movement potential and drainage, while nivation is linked to low radiation input on poleward-facing aspects. Our high-resolution modelling suggests that concurrently nearly half of the study region has a suitable climate for at least one of the LSP (Fig. 5a), reinforcing their importance as characterizing the geomorphology of cold-climate regions.

### Future periglacial climate realm

In Fig. 3 we contrast the future climates in the study area with the current periglacial realm and show the consistently shrinking area of overlapping envelopes (e.g., overlap with the baseline ca. 36% 2070–2099 RCP 4.5 and 11% 2070–2099 RCP 8.5). Our results show how even the operation of an optimistic emission pathway will initiate substantial alterations in the extent of the periglacial climate realm (Fig. 5b, c). For example, the suitable area for cryoturbation in our study area is predicted to shrink 84% (under RCP 2.6 by 2040–2069) compared to baseline. These changes in LSP distributions are driven by profound near-term changes in both increasing winter and summer air temperatures and precipitation in the study area (Supplementary Fig. 1). For example, the average (±s.d.) TDD in the study area was projected to increase from 1,117 °C (±323 °C; baseline) to 1,417 °C (±189 °C; 2040–2069 RCP 2.6) and to 1,645 °C (±417 °C; 2040–2069 RCP 8.5). Similarly, the amount of water precipitation is predicted to increase from 285 mm (±57 mm; baseline) to 350 mm (±35 mm; 2040–2069 RCP 2.6) and further to 387 mm (±82 mm; 2040–2069 RCP 8.5). Therefore, these predicted changes in temperature and precipitation regimes will cause the future periglacial realm to reduce markedly in size and the contemporary spatial extent of the periglacial realm will experience a climate that will be substantially warmer and wetter than present conditions (Supplementary Fig. 2).

The LSP loss will be enhanced in high-latitude low-relief continental areas, and in the Northern Hemisphere their projected future climate space is likely to contract dramatically as the Arctic Ocean will effectively limit their ‘‘range expansion’ northward^31^. Our modelling shows that with the highest greenhouse gas concentration scenario (RCP 8.5) no suitable climate exists for the development of palsas (permafrost mounding) by the end of this century, while any periglacial conditions are predicted to remain ~6% of the study area (Fig. 5c and Supplementary Table 1). Although our modelling does not account for lag times (i.e., the time difference from altered climate forcing to LSP response), the geographical changes are likely to be rapid because topmost soil layers are closely coupled with lower atmosphere conditions^14^. Despite a cover of insulating peat, many of the permanently frozen mires in the region formed during past cold climates are showing evidences of accelerated thawing^16, 32, 33^.

### Elevational shift

**Fig. 6:**
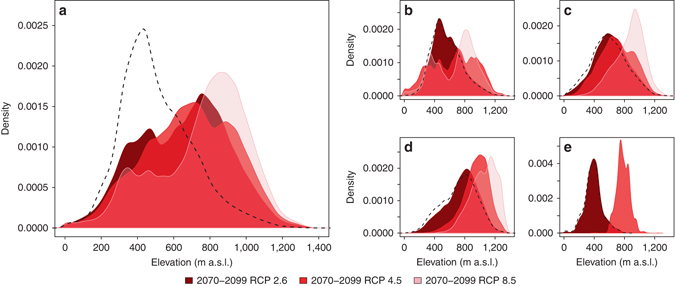
Shifting elevational distribution of the northern Europe’s periglacial climate realm. The kernel density plots (bandwidth=20) show the elevational distributions of the modelled LSP (**a** combined spatial extent, **b** cryoturbation, **c** gelifluction, **d** nivation and **e** permafrost mounding) under baseline 1981–2010 (*dashed black line*), 2070–2099 RCP 2.6, 2070–2099 RCP 4.5 and 2070–2099 RCP 8.5 climate conditions. For permafrost mounding (**e**), the kernel density estimation under scenario 2070–2099 RCP 8.5 was unfeasible due the low number of modelled occurrences. All distributions differed significantly (*t*-test, *p* ≤ 0.001)

In addition to a rapid decay of LSP, we predict significant elevational shift in periglacial conditions over the whole study domain (Fig. 6; *p* ≤ 0.001, *t*-test). The mean (±s.d.) elevation of the periglacial climate is projected to increase from 509 m above sea level (a.s.l.) (±199; baseline) to 650 m a.s.l. (±247; 2070–2099 RCP 2.6), to 686 m a.s.l. (±248; 2070–2099 RCP 4.5) and to 755 m a.s.l. (±252; 2070–2099 RCP 8.5). Consequently, our results suggest that the periglacial climate will be limited to high-elevation areas, after accounting for topographical and soil constraints. This means that suitable conditions for LSP at low elevations are the first to reduce, but potential shifts of LSPs to the highest mountains may be limited by steep topography and rocky or lack of frost-susceptible soil, although nivation processes may continue. These predicted elevational changes in the periglacial climate realm are likely to alter frost-driven denudation processes (e.g., slope processes and cryoplanation^1^) and modify decadal- to millennia-scale landscape development^11^. These climate changes will overwhelm frost as a geomorphic agent at low elevations, this being replaced by fluvial and aeolian activity. One consequence is that temperature- and precipitation-driven changes in perennial ground ice are likely to increase the risk of rapid slope displacements and thaw subsidence with pronounced societal impact in areas with infrastructure development^34^.

The reduction in the periglacial climate realm will trigger surface–atmosphere feedbacks with global relevance^7^. In periglacial environments as much as 90% of the total ecosystem carbon resides in frozen organic and mineral soils, and ground frost regimes are expected to form a major control on gas exchange processes (e.g., CO_2_ and CH_4_) between soil and atmosphere under climate change^8, 15, 16^. Changes in LSPs are likely to modify ground surface reflective properties by decreasing albedo through vegetation re-establishment^13^ with potentially significant implications for regional climate. Thawing permafrost in peat mounds will change local hydrology and enhance the formation of thermokarst lakes and ponds further decreasing albedo in a positive feedback loop^14^. Therefore, we stress the need for further (both spatially and temporally comprehensive) investigations of LSP–climate interactions.

Our approach for estimating the current and evolution of the future periglacial climate realm is based on statistical LSP–environment relationships^35^. While process-based Earth surface models with dynamical atmospheric components have been developed and applied over high-latitude regions, such process-orientated models require an intense parametrization, are computationally expensive and only provide coarse-scale projections. Moreover, process-based models do not provide spatially explicit prediction of LSP, rather they predict ground thermal regimes from which the inference of namely seasonal frost patterns can be challenging. We argue that scale is a critical issue in defining the periglacial climate realm, since (1) it should resemble the scale of observed LSP features (typically ranging from 1 m (a small polygon feature) to over 100 m (a permafrost peat plateau)) and (2) substantial local variation in LSPs driven by microclimate, topography and soil characteristics will be missed by coarse-scale modelling. This means that the extent of the current and projected future extent of the periglacial realm will be significantly underestimated. However, computational constraints means that modelling at such small spatial scales restricts the size of the region that can be examined, although we argue that our results (covering an area of 78,000 km^2^) are widely applicable to topographically and climatically similar Northern Hemisphere landscapes.

Our results are significant as they provide the first very fine-scale assessment of the current periglacial climate realm over a broad cold-region domain. Moreover, our findings suggest a near-complete decay of periglacial climate from a climatically sensitive high-latitude area and a significant elevational shift of cryogenic ground processes. Finally, these changes are likely to cause substantial landscape-scale changes in ground surface conditions, ecosystem functioning and biogeochemical cycles especially in high-latitude continental interiors. Our analysis, conducted over a wide range of future emission trajectories, indicates that regardless of the climate change mitigation policies the decay of periglacial system is likely to be rapid towards the end of this century.

## Methods

### LSP data

The study area lies between N 68–71° and E 20–26° and covers the transition from continuous to isolated permafrost, with strong temperature and precipitation gradients (from wet maritime to relatively dry continental) over tens of kilometres. According to a recent study^23^ continuous and discontinuous permafrost are limited to the highest mountains of the study area (ca. 6% and 18% of the observation sites with at least one LSP present, respectively), and thus the majority of the region can be classified as underlain by sporadic or isolated (50% and 25% of the observation sites, respectively) permafrost. This indicates that in large parts of the area LSPs are associated with seasonal freeze–thaw processes. The landscape of this climatically sensitive high-latitude region has been affected by multiple past glaciations. It includes the Scandes Mountains near the Arctic Ocean and low-relief areas to the south and east. The data comprise 2,917 study sites (25 × 25 m in size) and includes measurements of the surface features of four active cryogenic LSPs occurring in the area: cryoturbation, gelifluction, nivation and permafrost mounding. In brief, cryoturbation (i.e., frost churning) is a general term for soil movement caused by differential heave, and it creates typical periglacial surface features such as patterned ground and hummocky terrain^1, 36^. Gelifluction is a slow mass wasting process caused by high porewater pressure in unconsolidated surface debris where ‘downward percolation of water is limited by frozen ground and where melt of segregated ice lenses provides excess water which reduces internal friction and cohesion in the soil’, creating lobes and terraces^1^. Nivation is a collective term used to designate all aspects of weathering and fluvial processes, which are intensified and indicated by the presence of local snow accumulation sites^37^. Permafrost mounding creates mire complexes having a permanently frozen peat and mineral core (palsa)^10^.

The LSP sampling procedure is fully described in previous geomorphic studies^17^. As a summary, we used high-resolution aerial photography^38–40^ (spatial resolution of 0.25 m^−2^) and target field surveys to construct the LSP data set. A binary variable (1=presence, 0=absence) of each LSP was established indicating only the evident activity (or absence) of the LSPs. In a presence of a LSP, others were set as absent, although we are aware that some LSPs can overlap (e.g., a continuation from cryoturbation (mountain top) to gelifluction (slopes)). The data set does not include individually present periglacial microfeatures having a diameter of < 50 cm (e.g., mud boils, soil cracking due to frost action)^36^. The process was considered active if even a small area of the process had some indication of activity (absent vegetation cover, mixing of topmost soil, microtopography). The sampling covers the whole study domain and main climatological gradients. A random approach was not feasible because of the large size of the study area and inaccessible wetlands. To minimize uncertainties related to model extrapolation in time, the data sampling was extended ca. 100 km south of the prediction domain to cover the warmer temperature conditions that will potentially prevail at the prediction domain in the future.

### Background data

Monthly average temperatures (1981–2010, our baseline period) were modelled across the study domain (spatial resolution 50 × 50 m) based on 942 meteorological station (daily data from European Climate Assessment and Dataset^41^ (ECA&D)) and generalized additive modelling; as implemented in R-package mgcv^42^ version 1.8-7) utilizing variables of geographical location, topography (elevation, potential radiation, relative elevation) and water cover^43, 44^. In brief, our modelled monthly average air temperatures agreed well with the observations, with root mean squared error (RMSE) ranging from 0.56 to 1.58 °C. To obtain gridded precipitation data, a kriging interpolation was used based on the data from 1,076 rain gauges, topography and proximity to sea (R package gstat^45^ version 1.1-0). A random 10-fold cross-validation conducted over the gauge data indicated good agreement between measured and interpolated precipitation with RMSE ranging from 9.3 to 21.7 mm (Supplementary Fig. 3). Four climate variables with known physical relationship to the LSP activity^18, 23^ were calculated from the modelled climate data and used as predictors in LSP modelling: FDD (°C), TDD (°C), water precipitation and snow precipitation. TDD and FDD are based on the effective temperature sum of mean daily temperatures above or below 0 °C^46^, respectively. Thus, they provide an indication of frost intensity and melt period temperatures, being the factors most directly linked to cryogenic LSP^1, 20^. Water and snow precipitation represent accumulated rainfall (mm) with air temperatures above and below 0 °C, respectively. The consideration of snow precipitation is especially important due to its multiple effects on LSP due to insulation properties (constraining the development of permafrost peat mounds and cryoturbation) and snow accumulation (nivation)^47, 48^.

Three topographic variables were calculated from the digital elevation model (spatial resolution 50 × 50 m, data obtained from national land survey institutes of Finland, Sweden and Norway). These included slope angle (representing, e.g., mass movement potential), potential annual direct solar radiation^49^ (MJ cm^−2^ a^−1^; surface energy input) and topographic wetness index^50^ (TWI; a proxy for soil moisture). Two soil characteristic variables, rock (i.e., bare rock surfaces including, e.g., boulder fields and course surficial material) and peat cover, were extracted from a digital land cover classification^51^. Their effect on LSP derive from the thermal–hydrological properties of the soil, as peat has high moisture content and low thermal conductivity (except when frozen) and rock cover is associated with low moisture content and high thermal conductivity^1^. The original spatial resolution of the classification is 100 × 100 m, but the data were processed to a matching resolution of 50 × 50 m using nearest neighbour interpolation. Binary soil variables were transformed to a continuous scale using a spatial mean of 3 × 3 pixels representing the proportion of a soil variable within a modelling cell^17^.

### Climate model data

Climate projections for the twenty-first century are based on an ensemble of 23 global climate models (GCMs), derived from the Coupled Model Intercomparison Project phase 5 archive^29^. The data were processed to represent the predicted averaged changes in mean temperature and precipitation (in respect to the baseline 1981–2010) over two periods (2040–2069 and 2070–2099) and three RCP scenarios^28^ (RCP 2.6, RCP 4.5 and RCP 8.5). The climate model data depicting the predicted change in mean temperatures and precipitation respect to baseline climate were bilinearly interpolated to a matching resolution of 50 × 50 m and the predicted change by the GCMs was added to the spatially detailed baseline climate data. The four climate variables (TDD, FDD, water and snow precipitation) were recalculated for each time period and RCP scenario. Our results are consistent with climate model projections for high Northern latitudes, which tend to show Arctic amplification of surface and low troposphere temperatures^52, 53^.

### Statistical modelling

The occurrences of LSPs were related to the predictors using four statistical modelling techniques^35^: generalized linear modelling^54^, generalized additive modelling^55^, generalized boosting method^56^ and random forest^57^. All statistical methods are implemented in the Biomod2–platform^58^ (version 3.3-7) under R program^59^. The models were fitted using following specifications:

Occurrence of LSP=TDD+FDD+water precipitation+snow precipitation+radiation+slope angle+TWI+rock cover+peat cover.

Model performance was assessed with a repeated cross-validation scheme: the models were fitted 100 times by using a random sample of 70% of the data and subsequently evaluated against the remaining 30%. At each cross-validation run, the predicted and observed occurrences of LSPs were compared by calculating the area under the curve of a receiver operating characteristic plot^60^ and true skill statistics^61^ (TSS).

The models were used to forecast the LSP distributions in both baseline and future climates. The predicted probabilities of LSP occurrence were converted to binary presence–absence predictions according to TSS values calculated a priori during model evaluation (i.e., the TSS values were based on cross-validation statistics^61^). We constructed an ensemble of predictions using the majority vote approach^27^) where binary predictions are combined to a single agreement map. Here, a presence value for a given LSP inside each 50 × 50 m cell was denoted in the final map if three out of four modelling techniques voted (i.e., predicted) for its occurrence. Finally, for each time period and emission scenario, individual LSP predictions were summed up to show the number of overlapping LSPs at given cells.

### Data availability

Underlying data which support the findings of this study are available from the corresponding author on request.

## Electronic supplementary material

Supplementary Information

Peer Review File
