# Supplementary material for: Statistical modelling predicts almost complete loss of major periglacial processes in Northern Europe by 2100
Source: Nat Commun. 2017 Sep 11;8:515. doi: 10.1038/s41467-017-00669-3 (PMC5593823; doi:10.1038/s41467-017-00669-3)
Supplement: Supplementary file 1 — Supplementary Information [file 41467_2017_669_MOESM1_ESM.pdf]

Description of Supplementary Files

File Name: Supplementary Information
Description: Supplementary Figures and Supplementary Table

File Name: Peer Review File

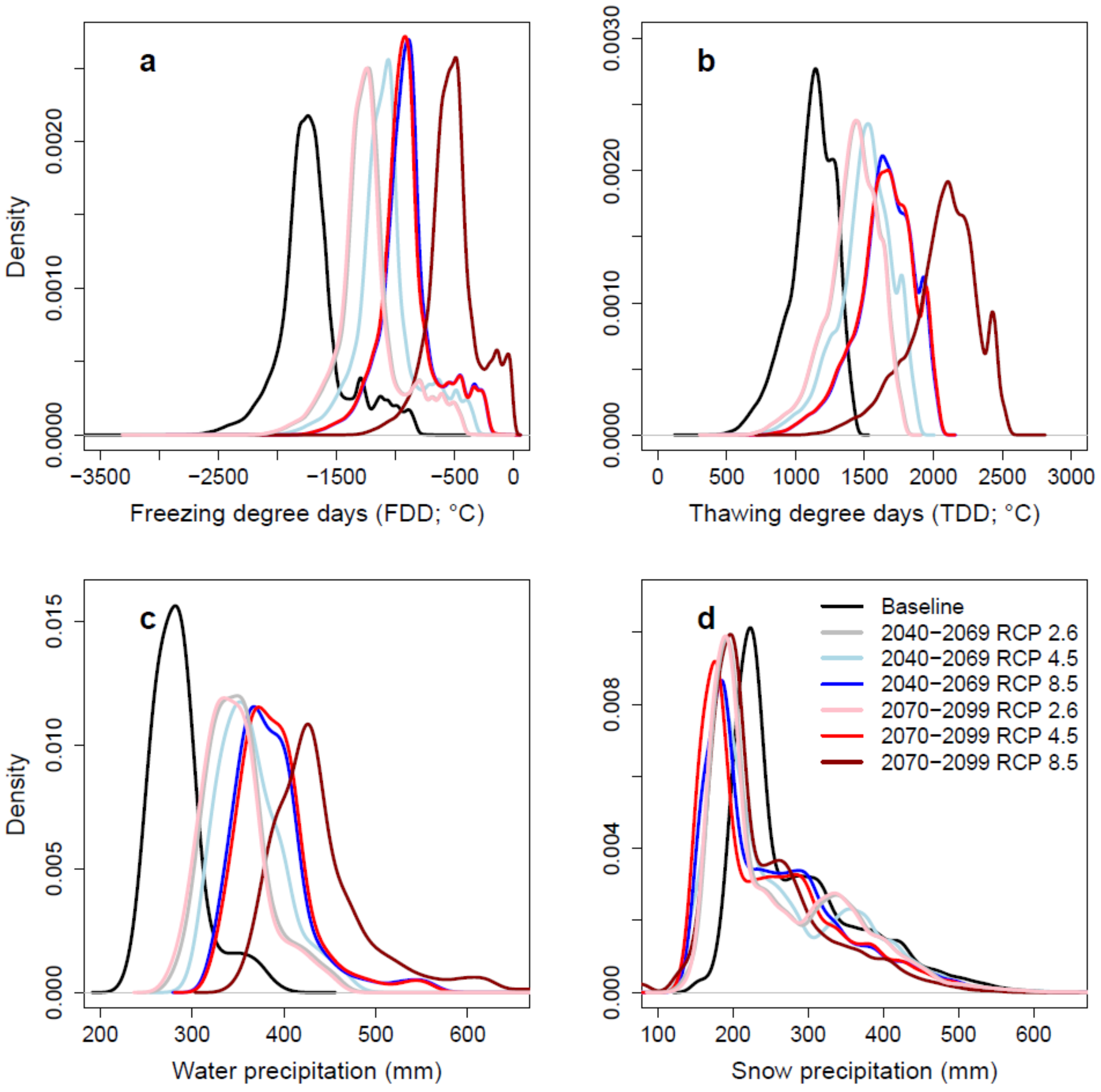

Supplementary Figure 1. Kernel density plots (bandwidth = 20 for TDD [**a**] and FDD [**b**], bandwidth = 10 for precipitation variables [**c** water precipitation, **d** snow precipitation]) showing the distributions of the four climate variables used for modelling LSP occurrence under three time slices and RCP scenarios. TDD = thawing degree days (°C), FDD = freezing degree days (°C).

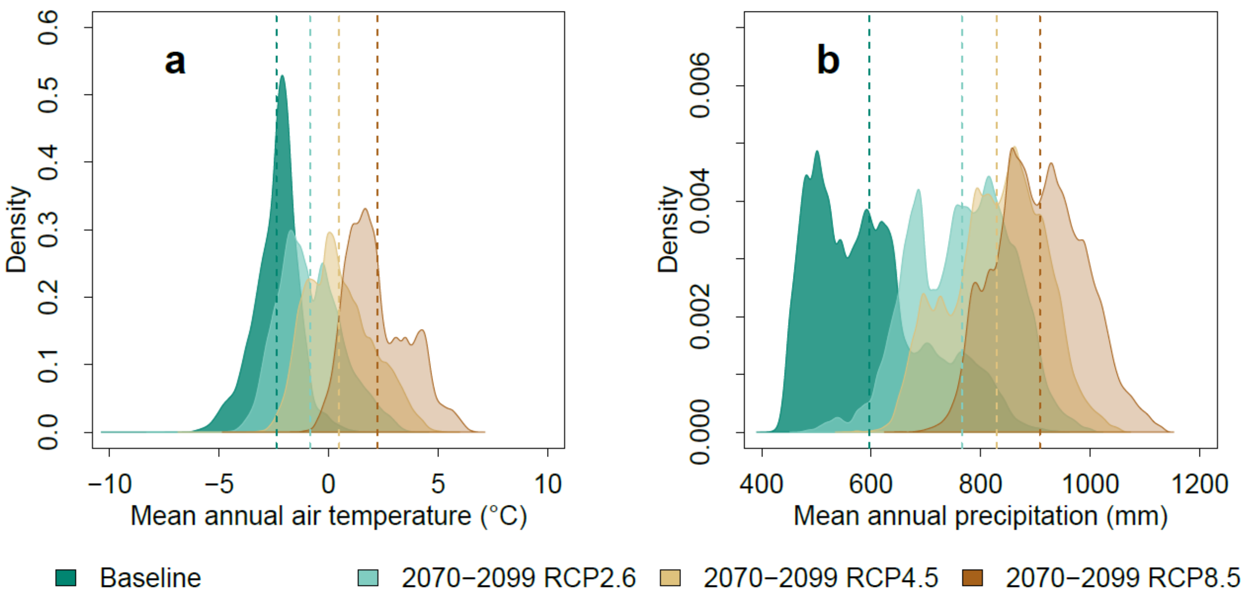

Supplementary Figure 2. The kernel density plots (bandwidth = 0.1 and 5 for mean annual air temperature [**a**] and precipitation [**b**], respectively) show the climatic distributions of the periglacial climate realm in the study area (i.e. combined spatial extent of LSPs) under baseline 1981-2010, 2070-2099 RCP 2.6, 2070-2099 RCP 4.5 and 2070-2099 RCP 8.5 climate conditions. The dashed lines depict the means of the non-smoothed distributions. All distributions differed significantly (t-test, $p \leq 0.001$).

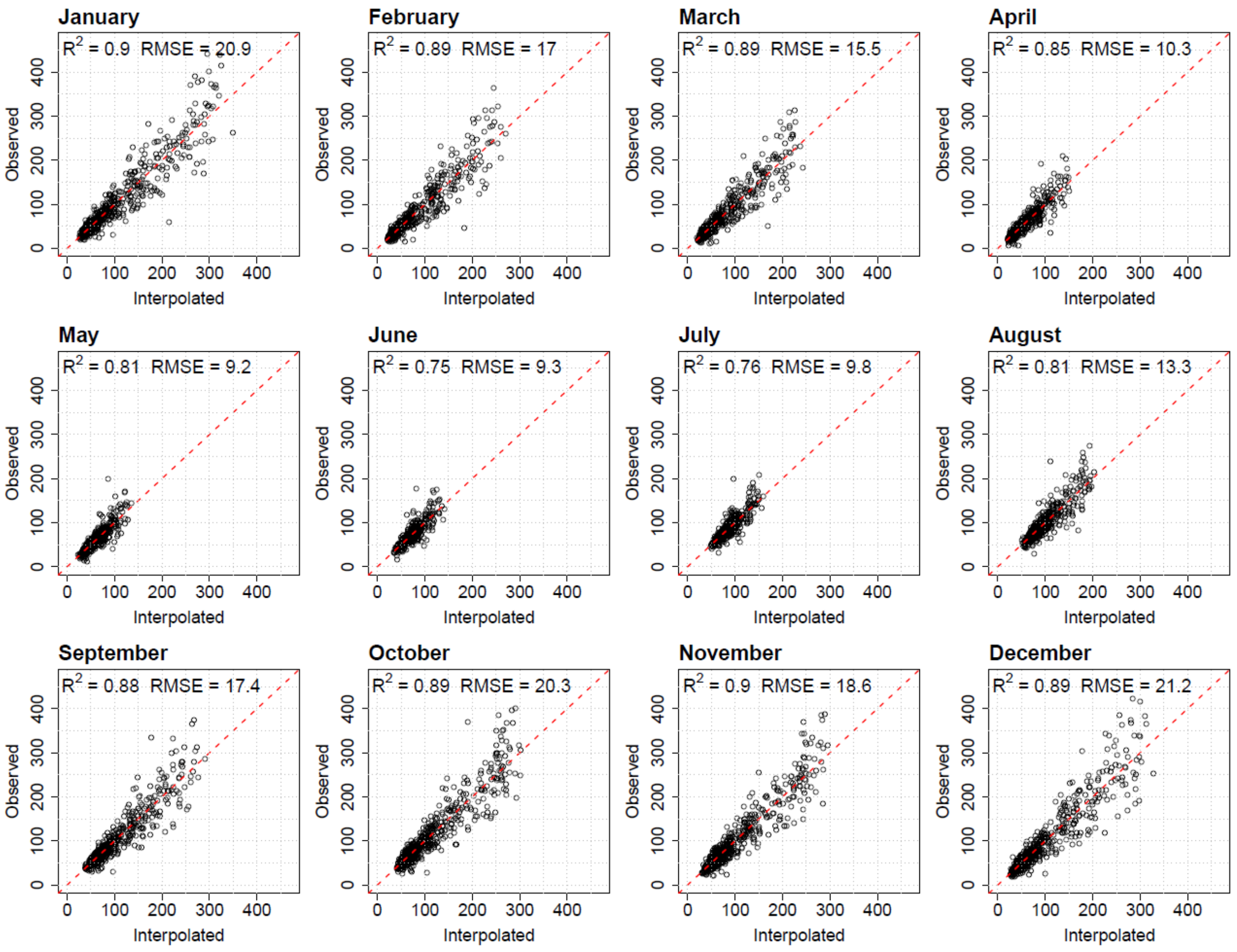

Supplementary Figure 3. The agreement between observed and interpolated monthly average precipitation sum (1981-2010, in mm) based on a ten-fold random cross-validation, in the terms of adjusted R-squared (R^2) and root mean squared error (RMSE). The red dashed line depicts 1:1 line.

Supplementary Table 1. The modelled covers (%) of overlapping climatic spaces suitable for cryogenic land surface processes (LSP) at the study area, over three time periods and climate change scenarios.

	**Baseline**	**RCP 2.6**	**RCP 4.5**	**RCP 8.5**	**RCP 2.6**	**RCP 4.5**	**RCP 8.5**
**Number of LSP**	1981–2010	2040–2069			2070–2099		
**1**	48.5	13.4	11.3	9.2	13.1	9.2	6.3
**2**	11.2	2.8	2.3	1.7	2.7	1.6	0.6
**3**	2.7	0.2	0.1	0.1	0.2	0.1	0.0
**4**	0.0	0.0	0.0	0.0	0.0	0.0	0.0
